# Authors’ response to letter “Prediction of acute kidney injury in intensive care unit patients”

**DOI:** 10.1186/s13054-019-2340-x

**Published:** 2019-02-19

**Authors:** Hiroyuki Naruse, Hiroshi Takahashi, Junnichi Ishii

**Affiliations:** 1 Department of Joint Research Laboratory of Clinical Medicine, Fujita Health University School of Medicine, 1-98 Kutsukake-cho, Dengakugakubo, Toyoake, 470-1192 Japan; 2 Division of Statistics, Fujita Health University School of Medicine, 1-98 Kutsukake-cho, Dengakugakubo, Toyoake, 470-1192 Japan

We thank Dr. Guo and coworkers for their interest and comments [1] on our article [2]. We have provided responses to their comments. First, we agree that the patient severity of illness and level of organ failure upon admission to medical cardiac intensive care units (MCICUs) may be important predictors for the development of acute kidney injury (AKI). Hence, we evaluated the predictive ability of urinary liver-type fatty acid-binding protein (L-FABP) and serum N-terminal pro-B-type natriuretic peptide (NT-proBNP) for AKI in the analytical model that included the Sequential Organ Failure Assessment (SOFA) score. In the multivariate logistic regression analysis, L-FABP, NT-proBNP, and the SOFA score were all independent predictors of AKI (Table 1). According to these findings, we speculate that a novel panel consisting of L-FABP, NT-proBNP, and the SOFA score may improve the accuracy for predicting AKI in patients treated in MCICUs. Furthermore, the addition of both L-FABP and NT-proBNP to a baseline model that included established risk factors and the SOFA score further enhanced the net reclassification and integrated discrimination improvement; this difference was greater than that obtained for either of the biomarkers and the baseline model alone (Table 2). Therefore, upon admission of patients to MCICUs, combining the measurements of the two independent predictors of AKI—L-FABP and NT-proBNP—may improve the accuracy for the early prediction of AKI beyond that achieved with either predictor alone.

**Table 1:** Multivariate logistic regression analyses for predictors of acute kidney injury

Variables	Multivariate model 1		Multivariate model 2	
	OR (95% CI)	*P* Value	OR (95% CI)	*P* value
Age (per 10 years increment)	1.18 (1.00–1.39)	0.05	1.21 (1.03–1.42)	0.02
IABP before admission	2.33 (1.32–4.10)	0.003	2.46 (1.42–4.27)	0.001
NT-proBNP (per 10-fold increment)	1.67 (1.22–2.29)	0.001		
Tertile of NT-proBNP (pg/mL)				
First (< 425)			1.0	
Second (425–2730)			2.10 (1.27–3.47)	0.004
Third (> 2730)			2.16 (1.20–3.88)	0.01
Urinary L-FABP (per 10-fold increment)	2.69 (2.06–3.50)	< 0.001		
Tertile of Urinary L-FABP (ng/mL)				
First (< 3.3)			1.0	
Second (3.3–11.5)			1.50 (0.92–2.44)	0.10
Third (> 11.5)			3.72 (2.34–5.93)	< 0.001
SOFA score (per 1 point increment)	1.12 (1.04–1.21)	0.004		
Tertile of SOFA score (point)				
First (< 2)			1.0	
Second (2–3)			1.17 (0.73–1.87)	0.51
Third (> 3)			2.04 (1.28–3.24)	0.003

Multivariate model adjusted for all baseline variables with *P* < 0.05 by univariate analysis. NT-proBNP, L-FABP, and the SOFA score were assessed as either continuous variables (model 1) or variables categorized into tertiles (model 2)

*CI* confidence interval, *IABP* intraaortic balloon pump, *L-FABP* liver-type fatty acid-binding protein, *NT-proBNP* N-terminal pro-B-type natriuretic peptide, *OR* odds ratio, *SOFA* Sequential Organ Failure Assessment

**Table 2:** Discrimination and reclassification of combination of L-FABP and NT-proBNP for acute kidney injury

	C-index	*P* value	NRI	*P* value	IDI	*P* value
Baseline model	0.752	Ref.		Ref.		Ref.
Baseline model + NT-proBNP	0.772	0.40	0.350	< 0.001	0.016	0.002
Baseline model + L-FABP	0.797	0.06	0.615	< 0.001	0.085	< 0.001
Baseline model + NT-proBNP + L-FABP	0.806	0.02	0.630	< 0.001	0.093	< 0.001
Baseline model + NT-proBNP + L-FABP vs. Baseline model + NT-proBNP	0.034*	0.14	0.571	< 0.001	0.077	< 0.001
Baseline model + NT-proBNP + L-FABP vs. Baseline model + L-FABP	0.008*	0.73	0.230	< 0.001	0.008	0.007

Baseline model included age, sex, hypertension, dyslipidemia, diabetes, smoking status, atrial fibrillation, acute decompensated heart failure, previous myocardial infarction, previous coronary revascularization, heart rate, emergent coronary angiography or percutaneous coronary intervention before admission, intraaortic balloon pump before admission, and the SOFA score

*IDI* integrated discrimination improvement, *L-FABP* liver-type fatty acid-binding protein, *NT-proBNP* N-terminal pro-B-type natriuretic peptide, *NRI* net reclassification improvement, *Ref.* reference

*Estimated differences between two groups

Second, unfortunately, the serum creatinine (SCr) concentration used for the diagnosis of AKI in our study had not been corrected according to fluid balance because of the inconsistent data recorded. Adjustment of the SCr concentration was proposed according to the assumption that the SCr concentration may be diluted by positive fluid balance [3]. However, Shen et al. have suggested that a large proportion of the infused fluid eventually leaks into the third space instead of contributing to blood volume [4]. Therefore, the use of adjusted SCr might have overestimated the AKI incidence [4]. Further studies are needed to clarify this issue.

Finally, the C-index observed following the addition of both L-FABP and NT-proBNP showed the improvement beyond that of the baseline model alone (Table 2). On performing the calibration using the Hosmer–Lemeshow test, the model involving the addition of both L-FABP and NT-proBNP to the baseline model showed a good fit, whereas the model involving the addition of a single biomarker or the baseline model alone showed a poor fit.

## Abbreviations

AKI: Acute kidney injury
L-FABP: Liver-type fatty acid-binding protein
MCICUs: Medical cardiac intensive care units
NT-proBNP: N-terminal pro-B-type natriuretic peptide
SCr: Serum creatinine
SOFA: Sequential Organ Failure Assessment
